# Heuristic Optimal Scheduling for Road Traffic Incident Detection Under Computational Constraints

**DOI:** 10.3390/s24227221

**Published:** 2024-11-12

**Authors:** Hao Wu, Jiahao Yang, Ming-Dong Yuan, Xin Li

**Affiliations:** 1 The Smart City Research Institute of China Electronics Technology Group Corporation, Shenzhen 518038, China; wuhao13@cetc.com.cn (H.W.); yangjiahao1@cetc.com.cn (J.Y.); 2 Guangdong Provincial Key Laboratory of Intelligent Urban Security Monitoring and Smart City Planning, Guangzhou 510200, China; 3 Faculty of Applied Sciences, Macao Polytechnic University, Macao 999078, China; xin.li@mpu.edu.mo

**Keywords:** traffic safety, intelligent monitoring, limited computational resources, heuristic optimal scheduling approach, event detection efficiency

## Abstract

The intelligent monitoring of road surveillance videos is a crucial tool for detecting and predicting traffic anomalies, swiftly identifying road safety risks, rapidly addressing potential hazards, and preventing accidents or secondary incidents. With the vast number of surveillance cameras in operation, conducting traditional real-time video analysis across all cameras at once requires substantial computational resources. Alternatively, methods that employ periodic camera patrol analysis frequently overlook a significant number of anomalous traffic events, thereby hindering the effectiveness of traffic event detection. To overcome these challenges, this paper introduces a heuristic optimal scheduling approach designed to enhance traffic event detection efficiency while operating within limited computational resources. This method leverages historical data and prior knowledge to compute a weighted event feature value for each camera, providing a quantitative measure of its detection efficiency. To optimize resource allocation, a cyclic elimination mechanism is implemented to exclude low-performing cameras, enabling the dynamic reallocation of resources to higher-performing cameras, thereby enhancing overall detection performance. Finally, the effectiveness of the proposed method is validated through a case study conducted in a representative region of a major metropolitan city in China. The results revealed a substantial improvement in traffic event detection efficiency, with increases of 40%, 28%, 17%, and 28% across different time periods when compared to the pre-optimized state. Furthermore, the proposed method outperformed existing resource scheduling algorithms in terms of average load degree, load balance degree, and higher computational resource utilization. By avoiding the common issues of resource wastage and insufficiency often found in static allocation models, this approach offers greater flexibility and adaptability in computational resource scheduling, thereby effectively addressing the practical demands of traffic anomaly detection and early warning systems.

## 1. Introduction

Despite advancements in transportation infrastructure, the risk of traffic incidents involving pedestrians, electric bicycles, and motor vehicles, which can lead to further traffic accidents, remains substantial on highways in developed countries. A study conducted in Shanghai, China, revealed that traffic accidents in the city have a high likelihood of resulting in secondary accidents ranging from 50% to 75% [1]. This alarming statistic underscores the urgent need for effective measures to mitigate the risks associated with traffic accidents. Rapid detection and elimination of traffic anomalies are critical to reducing accident rates, fatalities, and associated economic losses.

In this context, leveraging Internet of Things (IoT) technology has emerged as a critical approach to enhancing traffic safety. By integrating traffic detectors with IoT, a network of sensors can monitor and transmit real-time, precise data on traffic anomalies, enabling proactive traffic management. Beyond the rapid detection of anomalous behaviors, IoT technology empowers real-time data exchange and facilitates seamless integration with backend systems. This enables authorities to promptly respond to emerging traffic issues, ultimately contributing to a significant reduction in accident rates.

Consequently, traffic detectors are extensively employed to monitor road traffic conditions. These detectors promote traffic order and safety by collecting real-time data on various traffic elements, including pedestrians, vehicles, roadways, and environmental conditions. In addition, they gather behavioral and event data related to interactions between people and vehicles, as well as between vehicles and roadways, further contributing to effective traffic management [2].

Recent advancements in information technology, communication technology, sensor technology, and computer vision technology have spurred the evolution of traffic detectors toward greater diversity and intelligence. A diverse range of traffic detectors are employed to monitor road conditions, including loop, geomagnetic, video, microwave, infrared, and radar detectors. Among these, video detectors equipped with cameras strategically positioned on poles on both sides of the road can detect, identify, and track vehicles, providing valuable traffic data, such as vehicle type, speed, position, and traffic flow. Video detectors can also monitor traffic congestion and intelligently identify predefined abnormal events. Current research in video detection primarily focuses on the automatic recognition and early warning of these predefined anomalies or hazards, with significant efforts directed towards object detection and tracking [3,4], event recognition and semantic understanding [5], accident prediction and proactive response [6], and integrated intelligent traffic management [7].

With the construction and development of smart cities, video detectors are increasingly installed at urban traffic intersections and critical road segments. These detectors serve as invaluable auxiliary management tools, offering a high degree of deployment density and providing intuitive real-time insights for urban traffic management authorities. The increasing demands of urban management necessitate optimizing resources for monitoring road traffic, including surveillance cameras, to enhance road safety and traffic control efficiency. This challenge is particularly critical during emergency response situations, where timely and accurate detection of traffic incidents on major urban thoroughfares is essential to ensure public safety and facilitate rapid emergency response.

However, in practical engineering applications, two primary strategies are employed to detect traffic incidents within large-scale surveillance camera networks. The first approach entails concurrent operations across all cameras and incident types to enable real-time detection. Although effective, this method’s substantial computational requirements can significantly increase project costs. In contrast, the second approach uses a polling mechanism for cameras and incidents to detect traffic incidents. While the polling mechanism offers a computationally efficient alternative, it may compromise the detection of critical events, potentially hindering the overall effectiveness of traffic incident detection. Previous studies have extensively addressed strategies for intelligent placement of camera locations [8,9], the application of artificial intelligence algorithms [10,11], and the optimization of computational resource scheduling [12,13,14]. However, a notable gap exists in the literature regarding the intelligent selection of camera locations and the optimization of event detection scheduling within the context of constrained computational resources.

To address these challenges, this study introduces a heuristic optimization approach for traffic anomaly detection. This method maximizes detection efficiency while operating within constrained computational resources. Our approach involves randomly selecting a subset of cameras within a specified time window, leveraging prior knowledge to inform the selection process, and applying detection algorithms to each selected camera for all incident types. This approach ensures that all computational resources are fully utilized and operate at full capacity during each operational cycle. Following the detection phase, the weighted value of incidents for each event type within a cycle is calculated, ranked in descending order, and then the overall detection efficiency is obtained. Cameras with the lowest P% of weighted event feature values are discarded based on predefined rules. In the subsequent cycle, a random selection of cameras that have not yet been deployed for incident detection algorithms is made, ensuring that the freed-up computational resources are fully utilized. The overall detection efficiency of incidents is recalculated for each cycle. Based on the comparative analysis of efficiency between cycles, the decision to reiterate the steps is made. If a significant improvement is observed, the new configuration is retained. Conversely, if no improvement was detected, the process was repeated with a different arrangement. This iterative approach continues until an optimal configuration that maximizes overall event detection efficiency is identified. Finally, the effectiveness of the method is validated through a case study conducted in a typical area of a large metropolis in China. The results revealed a substantial improvement in traffic incident detection efficiency, with increases of 40%, 28%, 17%, and 28% across different time periods when compared to the pre-optimized state.

In summary, the contributions of this study are as follows:

1. This study introduces a heuristic optimal configuration and scheduling method for traffic anomaly detection, designed to maximize the efficiency of traffic event detection. By calculating the weighted event feature value for each camera to represent its detection efficiency, the method dynamically adjusts camera resource allocation based on detection requirements. This adaptive dynamic scheduling approach effectively addresses the limitations of static allocation models, avoiding resource wastage or insufficiency and enhancing overall detection performance.
2. An innovative cyclic elimination mechanism is introduced. This process identifies and removes low-performing cameras based on their detection efficiency within each cycle, and resources are redistributed from inactive cameras. This mechanism facilitates the continuous optimization of resource utilization across multiple cycles, thereby enhancing the flexibility and adaptability of computational resource scheduling.

The remainder of this paper is organized as follows: Section 1 introduces the adverse impacts of traffic incidents and existing problems related to current traffic incident detection. Section 2 reviews related studies on layout strategies for intelligent camera placement, the application of artificial intelligence algorithm optimization, and the optimization of computational resource scheduling. Section 3 presents the proposed method, followed by an empirical case study in Section 4. Finally, Section 5 provides a summary of the study.

## 2. Related Work

Previous studies on traffic incident detection have primarily centered on three key areas: intelligent monitoring point layout strategies, the application of artificial intelligence algorithm optimization, and the optimization of computational resource scheduling. This section provides an overview of relevant studies within these areas.

Regarding intelligent monitoring point layout, Xu et al. [8] conducted a review of the optimization layouts and design of road traffic detectors, considering factors such as detector types, application scenarios, data collection indicators, and the research objectives of various optimization studies. Micko et al. [15] addressed the challenge of optimizing monitoring tasks in intelligent transportation systems by developing a sensor classification framework that categorizes sensors according to their operating principles and maintenance requirements. Furthermore, they proposed a generic sensor system architecture designed to improve monitoring efficiency in intelligent transportation systems. Qiu et al. [16] simulated basic highway segments and scenarios involving abnormal events using a simulation platform. They employed an indirect event detection algorithm that identifies events based on changes in traffic flow parameters, in conjunction with direct perception of anomalies within the range of roadside detection equipment. This approach informed their determination of optimal video detector placement schemes and the associated benefits. Sun et al. [9] developed a multi-objective optimization model for traffic sensor layout, leveraging traffic big data to identify key influencing factors such as system cost, multi-source data sharing, data demand, sensor failure, road infrastructure, and sensor types. Similarly, Li et al. [17] proposed a multi-objective optimization framework to deploy traffic monitoring cameras on road networks, addressing comprehensive traffic management challenges such as accident prevention, traffic violation management, and traffic efficiency improvement.

In the context of artificial intelligence algorithm optimization, Bing et al. [18] studied how traffic flow parameters change both upstream and downstream of traffic incidents. Based on the analysis, an initial variable set with 12 variables to enhance traffic incident detection was developed. The random forest method was employed to screen key variables from the initial variable set. A relevant vector machine model with a combination kernel function was developed, and optimized through particle swarm optimization, to enhance the accuracy of traffic incident detection. Recent advancements in deep learning image processing and edge computing technology have significantly impacted the field of intelligent transportation. These developments were explored by Cao et al. [10], while Li et al. [19] addressed the challenges posed by small sample size and imbalanced datasets in traffic incident detection by introducing a hybrid detection method that leverages generative adversarial networks (GAN) and stacked autoencoders. Yan et al. [20] addressed the challenge of imbalanced datasets in video surveillance by introducing a semi-supervised approach to anomaly detection. Wan et al. [11] developed a long video event retrieval algorithm that utilizes superframe segmentation to eliminate redundant frames, significantly reducing the number of frames requiring subsequent computation. Singh et al. [21] introduced a novel framework for the automated detection of road traffic accidents in surveillance videos. In contrast to traditional handcrafted features, this framework utilizes a denoising autoencoder to automatically learn spatiotemporal feature representations from raw video data, extracting deep features and assessing the likelihood of accidents based on the reconstruction error and the probability of deep representations. Yan et al. [22] systematically analyzed the application of generative AI techniques in addressing critical challenges in traffic anomaly detection. Detecting unusual traffic events or patterns is essential for safety. By learning normal traffic patterns, generative AI models can effectively identify deviations as anomalies, leveraging techniques like conditional normalization of flow. Furthermore, these models can enhance sensor data quality by generating synthetic data that fill in gaps and provide a more comprehensive representation of traffic conditions. Qiu et al. [23] developed a vehicle recognition algorithm that utilizes convolutional neural networks enhanced by fused edge features (FE-CNN). This approach improves vehicle recognition accuracy and model convergence speed through a simple and efficient method of edge feature fusion. Li et al. [19] tackled the challenges of small sample size, imbalanced datasets, and real-time requirements in traffic incident detection by proposing a hybrid model designed specifically for this purpose. The proposed model employs GAN to increase the sample size and balance the dataset, while a Temporal-Spatio Stacked Autoencoder (TSSAE) is utilized to extract spatiotemporal correlations in traffic flow and identify potential incidents. Vishnu et al. [24] developed a cost-effective early warning system that integrates video processing technology with Vehicle-to-Infrastructure (V2I) communication. By using the Lucas–Kanade optical flow algorithm, the system can detect potential collision risks from moving objects and transmit timely alerts to vehicles. Ravishka et al. [25] introduced a traffic collision prevention system that leverages 5G/6G networks and edge computing to collect real-time vehicle location and dynamic data. By analyzing this information, the system can predict potential collisions and transmit early warnings to at-risk vehicles via low-latency communication.

In the area of computational resource optimization and scheduling, Ma et al. [12] proposed incorporating computational reuse technology into computational power networks to minimize service latency and optimize resource consumption by reusing the results of previous computational tasks. Jia et al. [13] proposed a deep integration of computational power with networks, facilitating the coordination of distributed computing resources to enhance overall computational resource utilization. Mijuskovic et al. [14] proposed a framework for evaluating resource management algorithms in cloud, fog, and edge environments. Their innovative approach provides valuable insights into the challenges and opportunities associated with resource allocation, workload balancing, resource provisioning, task scheduling, and quality of service (QoS). Lee et al. [26] developed a framework to enhance highway safety and efficiency by enabling real-time detection of critical traffic incidents and emergencies. The framework employs an enhanced ResNet classification algorithm integrated into an onboard module to monitor driving conditions and facilitate faster responses to hazardous events such as road debris, unexpected pedestrians, accidents, and vehicle breakdowns. Xu et al. [27] proposed an enhanced round-robin load balancing strategy that optimizes scheduling results by incorporating system state information. The scheduling involves a circular server list and a dynamic pointer that is adjusted based on the previously selected server, ensuring a more equitable distribution of tasks across resources. Kang et al. [28] introduced an adaptive scheduling algorithm based on minimum traffic, which assesses server load using network traffic parameters and establishes management thresholds to allocate new requests to low-load server nodes. Ge et al. [29] proposed TCL-ACO, an enhanced multi-objective ant colony optimization algorithm, tailored for cloud computing task scheduling. This approach seeks to simultaneously optimize task completion time, cost, and load balancing. Han et al. [30] introduced a multi-modal multi-objective particle swarm optimization algorithm (MMOPSOSS) equipped with an adaptive adjustment mechanism. This algorithm dynamically fine-tunes optimization parameters and population size to facilitate comprehensive convergence across multiple solution sets.

While previous research has largely concentrated on isolated aspects of smart city development, such as intelligent monitoring point placement or optimizing individual AI algorithms and computational resources, there remains a significant gap in exploring the integrated optimization of these elements within the context of specific business applications. This holistic approach is increasingly recognized as a crucial component of successful smart city initiatives. This study examines typical urban governance scenarios and road traffic safety management, investigating optimal automatic early warning capabilities for road traffic safety within the constraint of limited computational resources. Aiming to maximize the overall efficiency of traffic incident detection (OETID), specifically to detect as many critical incidents as possible, this study introduced a heuristic optimal configuration method for traffic anomaly detection. The following section offers a detailed introduction to this method.

## 3. Methodology

This section outlines the proposed method, which leverages historical data and prior knowledge, incorporating factors such as camera location selection, algorithm deployment, and deployment schedules to create a robust and intelligent system capable of effectively identifying and responding to critical traffic events. The method aims to optimize event detection capabilities within the constraint of limited computational resources to maximize OETID. To provide a clear and comprehensive understanding of our research, We will begin by defining key terms and concepts, followed by the proposal of a metric for OETID, and conclude with a detailed description of the method.

### 3.1. Preliminaries

Traffic incidents on highways are influenced by various factors, including human behavior, vehicular conditions, road conditions, and environmental influences. These incidents are characterized by sudden occurrence, random timing, and situation variability. Each traffic incident can be categorized based on its type and defined by three essential attributes: temporal, type, and spatial attributes.

- Temporal Attribute *t*: This refers to the specific time at which a traffic incident occurs. To facilitate data analysis, traffic incident data are often segmented into time windows *T*, that is, traffic incident data statistics are processed every *T* hour.
- Type Attribute *l*: This refers to the specific category of a traffic incident, such as pedestrians on highways, traffic congestion, and abnormal parking:
  (1) $$l\in \{l_{1},l_{2},\ldots ,l_{M}\},$$
  where *M* denotes the number of traffic incident types.
- Spatial Attribute *s*: This refers to the specific geographic location where a traffic incident occurs. In this study, the approximate location of the incident is determined by utilizing the geographic coordinates of the monitoring camera:
  (2) $$s\in \{s_{1},s_{2},\ldots ,s_{N}\},$$
  where *N* denotes the number of traffic surveillance cameras on the highway.

### 3.2. Overall Efficiency of Traffic Incident Detection

With limited computational resources, maximizing the utilization rate of computational resources can be achieved by deploying diverse detection algorithms across different cameras during specified time windows. This necessitates carefully determining the most suitable algorithm types for each traffic surveillance camera to maximize the collection of critical incident information within the available time frame, thereby maximizing the effectiveness of road safety accident prevention.

- To simplify the analysis, it is assumed, without loss of generality, that the computational cost of each traffic incident detection algorithm is the same across all cameras. This means that deploying any type of algorithm on any camera incurs the same computational overhead, denoted by *r*. Assuming that all cameras can deploy *M* types of incident detection algorithms, the maximum number of surveillance cameras that can be deployed using all *M* types of incident detection algorithms is *n* $\left(n=\frac{R}{M\times r},\;\text{where}\;R\;\mathrm{denotes}\;\mathrm{the}\;\mathrm{total}\;\mathrm{computational}\;\mathrm{resources}\right)$. In addition, a status value of 1 is assigned to surveillance cameras if they have deployed detection algorithms, while those without such algorithms are assigned a value of 0.
- Based on prior knowledge, such as business requirements or expert evaluation, the significance of the *j*-th type of incident is defined by $w_{j}$, where j=1,2,…,M; $d_{ij}$ denotes the number of *j*-type traffic incidents detected by the *i*-th camera.
- The weighted sum of the numbers of various abnormal incidents detected by each camera within a time window *T* is defined as follows:
  (3) $$f\left(i\right)=\sum_{j=1}^{m_{i}}w_{j}d_{ij},$$
  where $m_{i}$ denotes the number of algorithms deployed on the *i*-th camera and f(i) denotes the effective incident detection efficiency of the *i*-th camera.
- Furthermore, the average effective incident detection efficiency of all cameras with deployed algorithms is defined as the OETID for this time window and is calculated as follows:
  (4) $$f=\frac{\sum_{i=1}^{\beta }f\left(i\right)}{\beta }=\frac{\sum_{i=1}^{\beta }\sum_{j=1}^{m_{i}}w_{j}d_{ij}}{\beta },$$
  where β denotes the number of cameras. This study aims to maximize the OETID within computational constraints, with a specific focus on maximizing the average number of effective incident detections. To accomplish this objective, the following function can be derived:
  (5) $$\max f_{OETID}=\frac{\sum_{i=1}^{\beta }f\left(i\right)}{\beta },\;s.t.\sum_{i=1}^{\beta }m_{i}r\leq R.$$

### 3.3. Proposed Method

**Figure 1:**
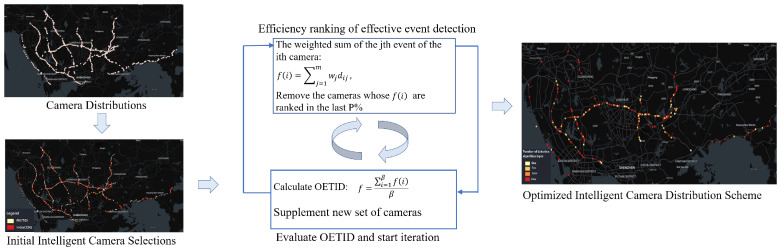
Schematic diagram of the traffic anomaly event detection efficiency optimization model workflow.

To optimize the overall efficiency of event detection within the constraints of limited computational resources, it is essential to continuously remove cameras with lower effective event detection efficiency according to predefined rules throughout the implementation of the method. In the subsequent cycle, the computational resources freed up by this process should be reallocated to new cameras for event detection, ensuring that all computational resources are fully utilized and operate at maximum capacity during each operational cycle. Consequently, this study focuses on optimizing the OETID following the removal of cameras with lower effective event detection efficiency, as illustrated in Figure 1.

Specifically, the pseudo code for this optimization model is illustrated in Algorithm 1 and primarily includes the following steps:

1. Set a time window *T* (e.g., 00:00–06:00). Based on prior knowledge or expert experience, heuristically and randomly select *n* cameras from the initial *N* cameras during the first round. Deploy all *M* types of detection algorithms on each selected camera, ensuring that all computational resources are fully allocated. This is calculated as: R=M×n×r. Additionally, mark all cameras that have been configured with algorithms as 1.
2. Calculate the effective event detection efficiency of each camera with deployed algorithms over a cycle (e.g., one week). For all cameras with deployed algorithms, if $d_{ij}=0$, it indicates that the *j*-th type of algorithm is no longer deployed on the *i*-th camera. Consequently, the freed-up computational resources can be reallocated to other relevant event types on different cameras, thereby maximizing the utilization of limited computational resources.
3. Sort the obtained effective event detection efficiencies in descending order. Remove the cameras corresponding to the bottom P% (where *P* denotes a specified value, typically in the range (0, 20]), and calculate the remaining computational resources $R_{s}=R-\sum_{i=1}^{n^{\prime }}r\times m_{i}$, where $n^{\prime }$ denotes the number of cameras with the deployed detection algorithms. Furthermore, calculate the OETID for the top 1−P% cameras.
4. Enter the next cycle. Select $\left\lceil R_{s}/M\right\rceil$ cameras (where ⌈.⌉ denotes the ceiling function) from those marked as 0, and deploy all *M* types of detection algorithms on each selected camera to ensure that all remaining computational resources are fully allocated. Mark these cameras as 1. Use steps (2) and (3) to calculate the OETID $f_{C}$ for the top 1−P% cameras in this cycle (where $f_{C}$ denotes the OETID for cycle *C*).
5. If the designated number of cycles is reached or all cameras are marked as 1, complete the optimization, exit the loop, and terminate the iteration. After the algorithm iteration is concluded, sort the obtained weighted event feature values in descending order and remove the cameras corresponding to the bottom P% of effective event detection efficiency. To maximize the utilization of computational resources, the saved computational resources can be dynamically allocated by relevant personnel based on experience and urgency of tasks.
6. In the next time window *T* (e.g., 06:00–12:00), repeat steps (1)–(5) until all time windows *T* are fully optimized.

**Algorithm 1** Pseudo Code of Dynamic Computing Resource Allocation  1:**Input:** *T*, *N*, *n*, *M*, *r*, *P*, β, maxcycles  2:**Parameter:** *R*, Rs, status, *d*, *f*, f_sort, *w*, $m_{i}$  3:**Output:** resource_allocation_plan  4:Initialize status←[0]×N  5:select_random_cameras(n)_for_status==1  6:R←M×n×r  7:Rs←0  8:$m_{i}\leftarrow M$  9:cycle←010:$maxcycles\leftarrow \frac{24}{T}$11:**while** cycle<maxcycles **do**12:      **while** any(status==0) **do**13:            Deploy_cameras_plan14:            **for** i←1 to *n* **do**15:                  **if** status[i]==1 **then**16:                        **for** j←1 to $m_{i}$ **do**17:                              **if** d[i][j]==0 **then**18:                                   $remove\_algorithm\_d_{\left(i,j\right)}$19:                                   $m_{i}\leftarrow m_{i}-1$20:                              **end if**21:                        **end for**22:                  **end if**23:            **end for**24:            **for** i←1 to *n* **do**25:                  $f\left(i\right)\leftarrow \sum_{j=1}^{m_{i}}w_{j}\cdot d_{ij}$26:            **end for**27:            f_sort←sort(f(i))28:            num_to_remove←P×len(f_sort)//10029:            $n^{\prime }\leftarrow num\_to\_remain=n-num\_to\_remove$30:            **for** i←1 to num_to_remain **do**31:                  camera_to_remain←f_sort[i]32:                  $f1\leftarrow \frac{\sum_{i=1}^{n^{\prime }}w_{j}f\left(i\right)}{n^{\prime }}$33:            **end for**34:            **for** i←n−num_to_remove+1 to *n* **do**35:                  camera_to_remove←f_sort[i]36:            **end for**37:            $Rs\leftarrow R-\sum_{i=1}^{n^{\prime }}r\cdot m_{i}$38:            $num\_new\_cameras\leftarrow \frac{Rs}{M}$39:            **Select** num_new_cameras **from** unallocated_cameras40:            **for** i←1 to num_new_cameras **do**41:                  status[camera_to_new[i]]←142:            **end for**43:      **end while**44:      $f\leftarrow \frac{\sum_{i=1}^{\beta }f\left(i\right)}{\beta }$45:      **for** i←1 to *N* **do**46:            status[i]←047:      **end for**48:**end while**49:**return** resource_allocation_plan

## 4. Experimental Analysis

### 4.1. Data and Processing

This section examines a megacity in China, focusing on its highway and urban expressway, to optimize and validate the existing road traffic incident detection scheme using the proposed method. To enhance data reliability and mitigate the impact of sporadic traffic incidents, a 14-day cycle was used for statistical analysis of the data. This cycle serves as the basis for optimizing resource allocation under limited computational resources.

The data used in this study include traffic surveillance camera location data and traffic incident data. The traffic surveillance camera location data were sourced from the deployment information provided by the traffic management department, including the identification (ID) codes and the latitude and longitude coordinates of each surveillance camera. The traffic incident data were sourced from the incident detection system of the traffic management department. These data include the ID codes of the surveillance cameras with deployed intelligent algorithms, the types of automatically detected incidents, detection times, and the resolution status of each incident.

To ensure the reliability of the computational results, a thorough data preprocessing process was conducted. First, the traffic surveillance camera location data were linked to the traffic incident data using camera ID codes. This integration allowed for the approximation of the geographic locations of traffic incidents based on the locations of the corresponding surveillance cameras. Next, traffic incidents that were manually confirmed as false alarms were filtered out to ensure a valid dataset. Finally, statistical analysis was conducted on the number and types of incidents, categorized by different times, intervals, and regions.

**Figure 2:**
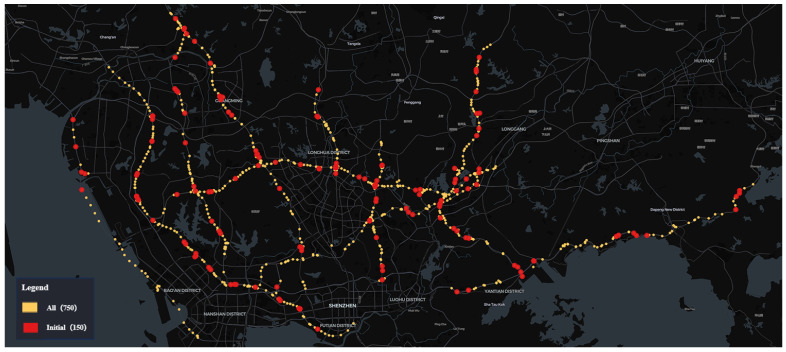
Spatial distribution characteristics of the 150 cameras in the initial state.

The city has a total of 750 traffic surveillance cameras installed across a specific administrative district’s expressways, monitoring key traffic events that affect road safety, such as pedestrians on highways, abnormal parking, congestion, and non-motorized vehicles on highways. The computational resource *R* is sufficient to deploy four types of traffic event detection algorithms on 150 traffic surveillance cameras simultaneously. The dataset utilized for this analysis comprises traffic event data collected by these 150 cameras within 6 h time windows *T*, spanning the period from 1 July 2023 to 6 April 2024. These 150 cameras were selected by the traffic management department based on their expertise and experience in accident prevention. The spatial distribution of these cameras across the expressway network is shown in Figure 2.

### 4.2. Data Evaluation Metrics

To comprehensively and accurately evaluate the actual performance of the proposed method and gain a deeper analysis of its scheduling effects, this study employs computational resource utilization, average load degree, and load balance degree as the core evaluation metrics.

Computational resource utilization refers to the ratio of the actual computational power used by the system to the total available computational power. This metric reflects the effectiveness of resource utilization within the system. The calculation formula is as follows:

(6) $$U=\frac{\sum_{i=1}^{N}R_{i}}{R}\times 100\%$$

where *U* is the computational resource utilization, $R_{i}$ ($R_{i}=m_{i}\times r$) represents the load (or computational power consumption) of the *i*-th camera, and *R* is the maximum computational capacity. Computational resource utilization measures the effectiveness of resource allocation within the system. A utilization rate approaching 100% signifies optimal resource allocation, with no idle resources. Conversely, a lower utilization rate may suggest resource waste or improper scheduling within the system.

The average load degree represents the overall load distribution across all cameras. It is calculated as the mean of the individual camera loads, where load can be defined in terms of computational power consumption, workload, or event detection workload of each camera. The calculation formula is as follows:

(7) $$\bar{R}=\frac{1}{N}\sum_{i=1}^{N}R_{i}$$

where $\bar{R}$ represents the average load degree. The average load degree reflects the overall load condition of the system. A high average load degree signifies that most cameras are operating under heavy load, while a low average load degree suggests that most cameras have lighter loads, potentially indicating idle resources within the system.

The load balance degree is evaluated using the standard deviation, which measures the degree of uneven load distribution among the cameras in the system. It reflects the disparity in load between different cameras and quantifies the imbalance in load distribution among the cameras. The calculation formula is as follows:

(8) $$\sigma =\sqrt{\frac{1}{N}\sum_{i=1}^{N}{\left(R_{i}-\bar{R}\right)}^{2}}$$

where σ represents the load balance degree. The load balance degree measures the unevenness of workload distribution among the cameras in the system. A high load balance degree indicates a significant disparity in load, with some cameras operating under heavy load while others have lighter workloads. This imbalance can lead to processing bottlenecks on the overloaded cameras, affecting the overall system efficiency and stability. Conversely, a lower load balance typically signifies a more even distribution of load, which is a sign of effective load distribution across the cameras. This indicates effective load optimization and suggests that the system’s overall performance is at its best.

### 4.3. Experimental Results and Analysis

In practice, the selection of the 150 traffic surveillance camera locations was based on experience, coupled with the sporadic nature of abnormal events. As a result, it is uncertain whether this configuration scheme will achieve the highest detection efficiency for incident instances. Therefore, this configuration is regarded as the initial scheme, and an optimization method will be applied to enhance the configuration according to the aforementioned approach. Weights are assigned based on expert experience and are objectively determined according to historical incident statistics and the societal impact of these incidents. This approach results in a comprehensive score, ensuring that the sum of all event weights equals 1. Table 1 shows the weight values for the four types of traffic incidents: pedestrians on highways, abnormal parking, traffic congestion, and non-motorized vehicles on highways.

Using the initial data from 1 July 2023 to 14 July 2023 and applying Equations (3) and (4), traffic incident data and detection efficiency are calculated from the 150 traffic surveillance cameras equipped with detection algorithms for each time window. The collected traffic incident data and OETID values under this scheme are presented in Table 2.

**Figure 3:**
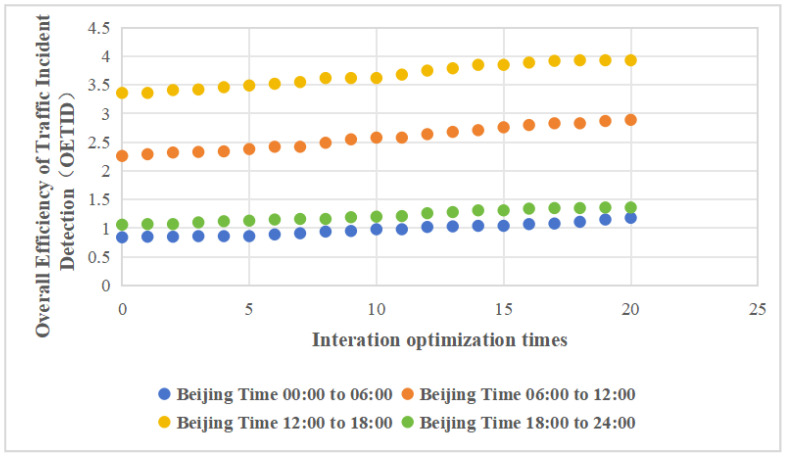
The values of the overall efficiency of traffic incident detection with iterations.

Based on the optimization method, the effective event detection efficiency f(i) within each time window is ranked, and the cameras corresponding to the bottom 20% of incident cases are removed. Additionally, following the computational resource allocation principle, if the number of *j*-type traffic incidents detected by the *i*-th camera is 0, the algorithms for that type of incident will no longer be deployed at that location. The remaining computational resources are reallocated to establish the optimal detection configuration scheme. The optimization method is applied to calculate the optimal detection configuration scheme for each of the four time windows separately. As shown in Figure 3, after 20 iterations, all camera configurations are completed. This figure illustrates the following observations:

1. With each successive iteration, the OETID within the four time windows demonstrated steady improvement. This indicates that continuous optimization iterations can enhance the identification of traffic incidents, even under the constraints of limited computational resources.
2. Throughout the iterative optimization process, the rankings of detection efficiency for the four time windows consistently remain in descending order: 12:00–18:00, 06:00–12:00, 18:00–24:00, and 00:00–06:00. The detection efficiency during the time window 12:00–18:00 is significantly higher, while the efficiency during time windows 18:00–24:00 and 00:00–06:00 are relatively similar. This indicates that the efficiency of traffic incident detection is greatly influenced by the time of day, underscoring the need for an optimization method tailored to different times.

The OETID under the initial and optimal configuration schemes for the four time windows is presented in Table 3.

As illustrated in the figure, the optimized detection efficiency improves across the four time windows. Notably, during the 00:00–06:00 time window, detection efficiency increased from 0.84 to 1.18, representing a 40% improvement. In the 06:00–12:00 time window, detection efficiency increased from 2.26 to 2.89, resulting in a 28% improvement. Similarly, in the 12:00–18:00 time window, detection efficiency increased from 3.36 to 3.93, showing an improvement of 17%. Lastly, in the 18:00–24:00 time window, detection efficiency increased from 1.06 to 1.36, showing an improvement of 28%.

**Figure 4:**
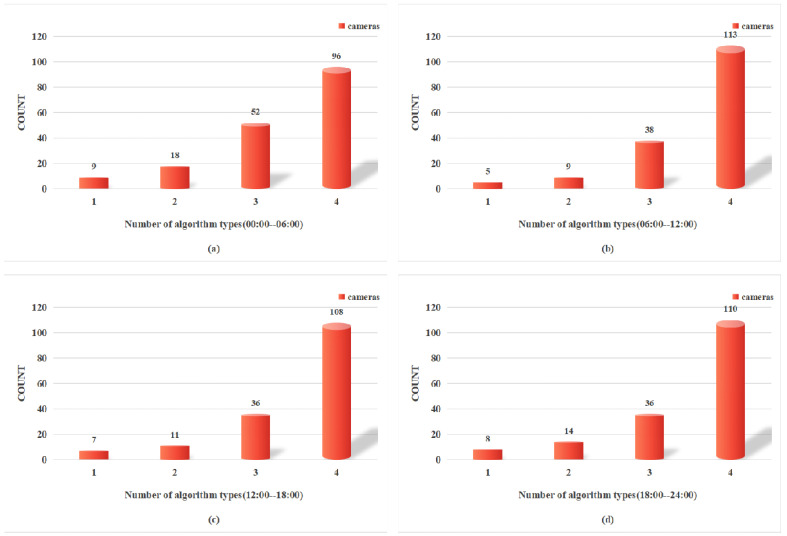
Number of algorithm types deployed on cameras in each time window.

Due to limited computational resources, there is a notable disparity in the number of algorithm types deployed on the cameras across each time window. In the 00:00–06:00 time window, as shown in Figure 4a, there are 175 camera locations with at least one type of algorithm deployed. Among these, 96 cameras are equipped with all types of algorithms, while 9 cameras utilize only one type of algorithm. In the 06:00–12:00 time window, as illustrated in Figure 4b, 165 camera locations had at least one type of algorithm deployed. Among these, 113 cameras were configured with all types of algorithms, while only 5 cameras had a single type of algorithm deployed. In the 12:00–18:00 time window, as shown in Figure 4c, 162 camera locations had at least one algorithm deployed, with 108 cameras utilizing all types and 7 cameras using only one. Finally, in the 18:00–24:00 time window, as shown in Figure 4d, 168 cameras have at least one algorithm deployed, with 110 cameras using all algorithm types and 8 cameras using only one.

The overall performance indicators for traffic events during the four time periods are presented in Table 4. The highest overall detection efficiency is achieved during the 12:00–18:00 time window, indicating that the cameras perform most effectively during this period. In contrast, the lowest efficiency is observed between 00:00 and 06:00, possibly due to reduced monitoring activities during these hours. The average load degree shows minimal variation across the time periods, indicating a relatively stable overall trend. This consistency suggests that the system applies a balanced load scheduling strategy to ensure that the computational task load of each camera remains relatively consistent across different time periods. Regarding the load balance degree, the time window from 00:00 to 06:00 experiences lower traffic flow and fewer monitoring tasks, resulting in a lower load balance degree. In the 06:00–12:00 time window, corresponding to the morning rush hour (06:00–09:30), traffic flow increases significantly, and traffic events occur more frequently, causing noticeable discrepancies in the load across different cameras. Similarly, the 12:00 to 18:00 window, which includes both the noon and evening peak hours (11:30–13:30 and 17:00–20:00), sees substantial traffic flow and complex incident patterns, particularly on major roads, leading to higher loads across the cameras. However, non-major roads experience relatively lighter traffic, which exacerbates the issue of load imbalance. During the 18:00–24:00 time window, which corresponds to the late stage of the evening peak (19:00–20:00), traffic flow and the occurrence of incidents gradually decrease. As a result, the load differences among cameras become less pronounced, reflecting a more balanced distribution of computational tasks. Computational resource utilization shows minimal variation across the time windows, demonstrating the effectiveness of the dynamic resource scheduling mechanism. The monitoring system maintains a substantial reserve of computational resources, enabling a balanced distribution during different time periods and ensuring a consistently high level of computational resource utilization.

**Figure 5:**
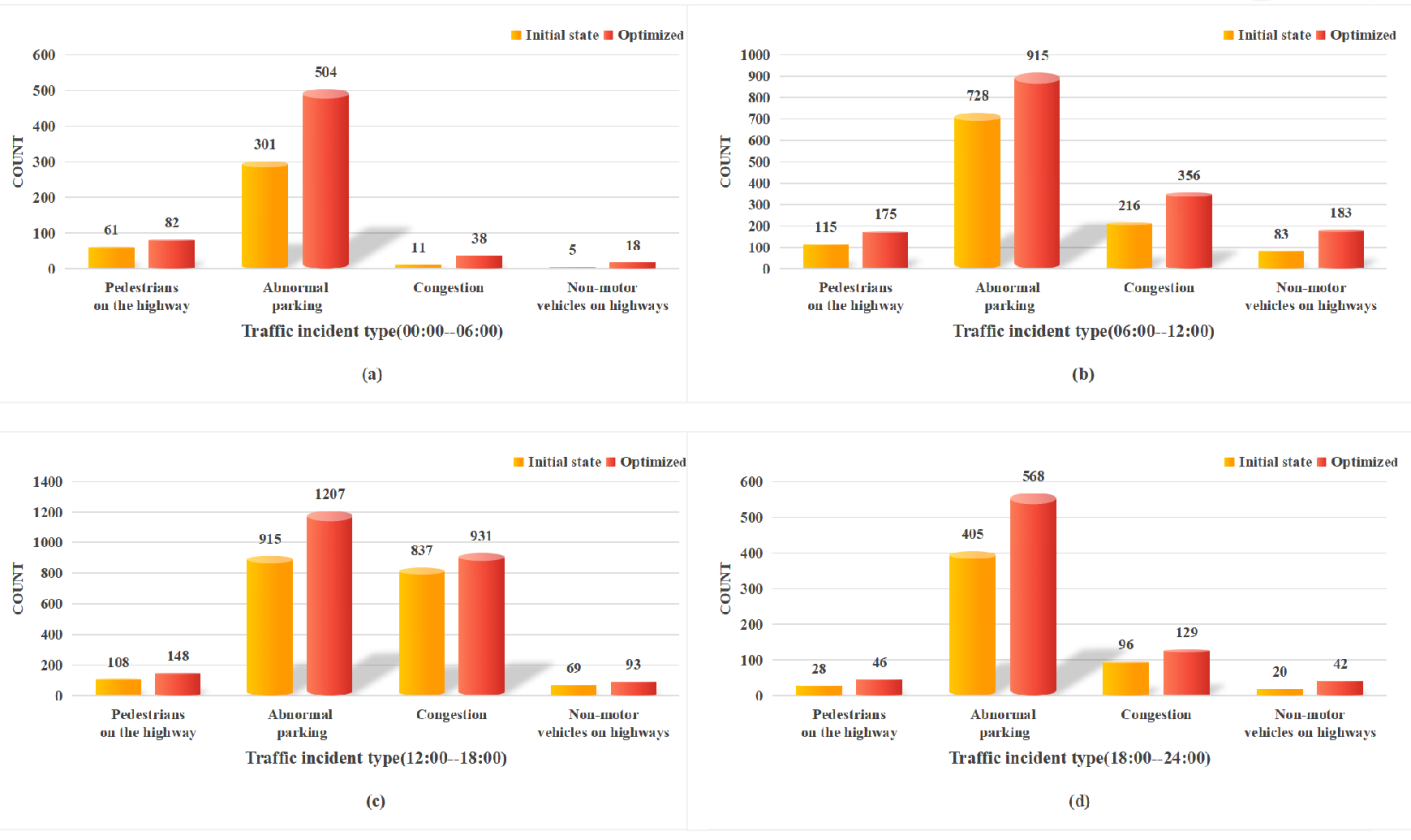
Comparison of the number of detected incidents by type before and after optimization across different time windows.

In terms of the number of incidents detected, the optimized scheme has increased the number of detected incidents for all types of traffic events, as illustrated in Figure 5a–d, which illustrate the time windows 00:00–06:00, 06:00–12:00, 12:00–18:00, and 18:00–24:00, respectively. Overall, the distribution of incident types within each time window exhibits both similarities and differences.

The similarity across all time windows is that abnormal parking consistently emerges as the most frequent and significantly increasing incident type. This trend highlights the widespread nature and severity of abnormal parking issues in traffic incidents. Regardless of the time, addressing abnormal parking remains a critical priority for effective traffic management and urban planning.

A notable difference occurs in the time window 12:00–18:00. While abnormal parking remains a prevalent issue, the number of congestion incidents is relatively high during this period. This reflects the increased volume of traffic and its contribution to road congestion. Moreover, the prevalence of abnormal parking incidents can further intensify traffic congestion, because inappropriate parking behavior occupies road space and disrupts traffic flow. To effectively manage traffic and enhance road efficiency, authorities must prioritize addressing both congestion and abnormal parking issues.

Commonly used methods for scheduling computing power in event detection include static round-robin scheduling, weighted round-robin scheduling, and priority round-robin scheduling. Static round-robin scheduling distributes computing power evenly among all cameras, allocating resources in a rotating manner at fixed time intervals, ensuring that each camera receives an equal amount of computing power. Weighted round-robin scheduling assigns a fixed weight to each camera based on its actual operational capacity. When it is a camera’s turn to receive resources at predetermined intervals, allocations are made in proportion to its assigned weight. Priority round-robin scheduling allocates computing power based on the priority assigned to each camera, ensuring that higher-priority cameras receive computing resources first. In this study, the proposed heuristic adaptive scheduling method is compared with the three widely adopted methods using the same dataset. The comparison of different traffic event detection computing power scheduling methods is presented in Table 5, where each of the three values is derived from the average across four time windows.

In static round-robin scheduling, the lack of consideration for the actual load demands of the cameras can lead to inefficiencies. Cameras with lighter loads may waste computing power, while those with heavier loads may not receive sufficient resources, resulting in significant load disparities among the cameras. As a result, the average load degree is lower and the load balance degree is higher, reaching values of 4.25 and 37.43, respectively. However, the computational resource utilization is lower at only 77.22%. In comparison to static round-robin scheduling, the proposed method demonstrates significant performance improvements. The average load degree and load balance degree are optimized by 62% and 47%, respectively, indicating a more equitable distribution of workload among the cameras. Additionally, the computational resource utilization is increased by 26%, suggesting more efficient use of resources. 

In weighted round-robin scheduling, the method does not dynamically adapt to fluctuations in load and instead relies on initially assigned weights based on anticipated camera capabilities. Under typical conditions, the load degree of the cameras aligns more closely with actual demands, yielding performance similar to the method proposed in this paper. However, when low-weight cameras encounter unexpected traffic events, they may not have adequate computing power. In comparison to the proposed method, weighted round-robin scheduling exhibits slightly lower performance, reaching values of 5.78 and 25.32 in average load degree and load balance degree, respectively. The computational resource utilization is slightly lower at 89.67%. In comparison to weighted round-robin scheduling, the proposed method demonstrates improvements in all three performance indicators: average load degree, load balance degree, and computational resource utilization. The proposed method optimizes these indicators by 19%, 22%, and 8%, respectively. 

In priority round-robin scheduling, this approach often leads to insufficient computing power for lower-priority cameras, further exacerbating disparities among cameras. As a result, this method exhibits a lower average load degree and a higher load balance degree, reaching values of 5.52 and 29.65, respectively, while the computational resource utilization is reduced to 85.34%. Compared to priority-based round-robin scheduling, the method proposed in this paper optimizes the three indicators by 25%, 33%, and 14%, respectively. 

In summary, the heuristic adaptive scheduling method proposed in this paper demonstrates a higher average load degree, reduced load imbalance degree, and enhanced computational resource utilization. This approach effectively mitigates issues associated with inefficient resource allocation, resource wastage, and insufficient computing power that are common in static round-robin scheduling, weighted round-robin scheduling, and priority round-robin scheduling. This approach enables dynamic resource allocation and promotes efficient utilization of camera resources.

**Figure 6:**
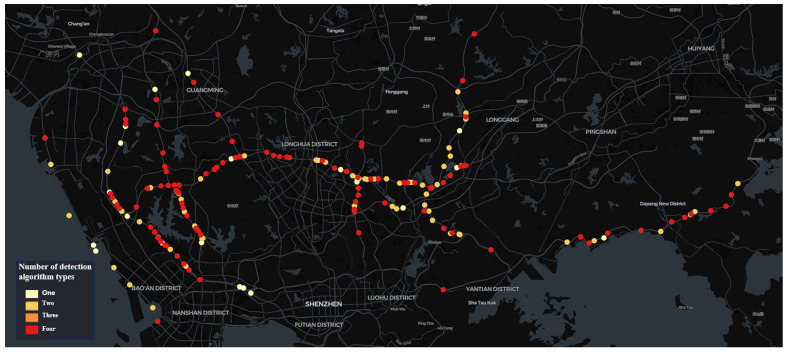
Spatial distribution characteristics of 175 intelligent detection cameras after optimization for the time window 00:00–06:00.

**Figure 7:**
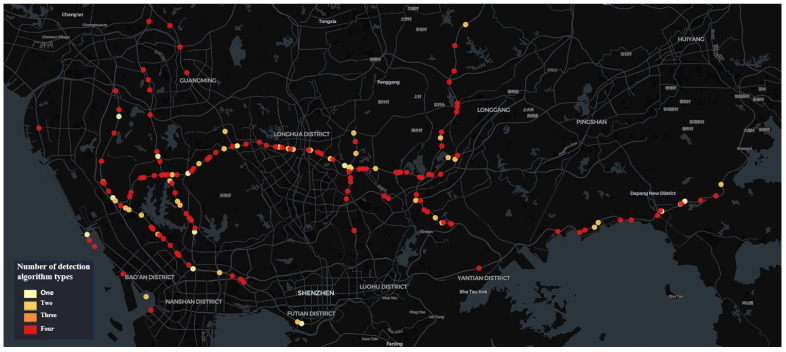
Spatial distribution characteristics of 165 intelligent detection cameras after optimization for the time window 06:00–12:00.

**Figure 8:**
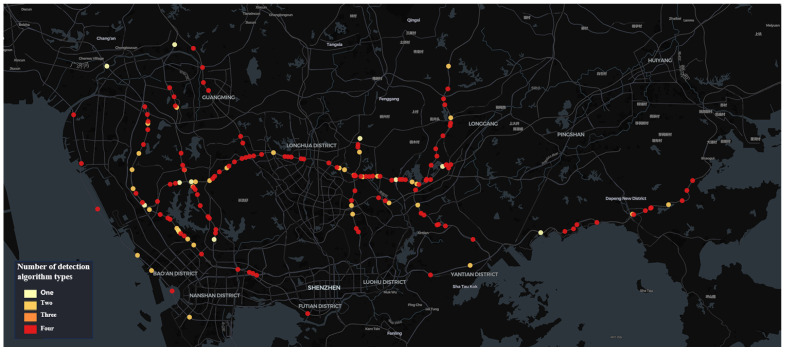
Spatial distribution characteristics of 162 intelligent detection cameras after optimization for the time window 12:00–18:00.

**Figure 9:**
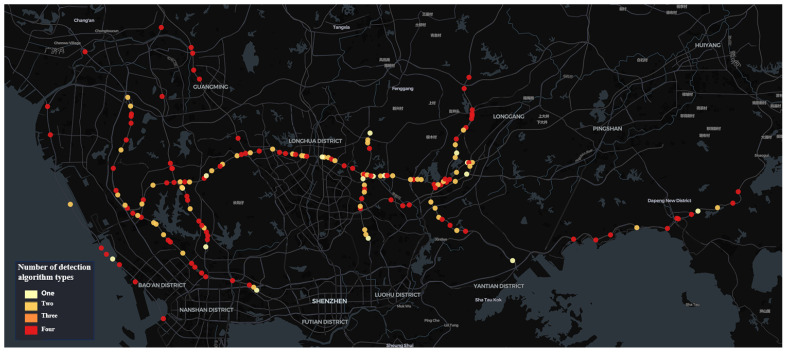
Spatial distribution characteristics of 168 intelligent detection cameras after optimization for the time window 18:00–24:00.

As can be observed from the results of the camera distribution (Figure 6, Figure 7, Figure 8 and Figure 9), after optimization, the camera locations within each time window are more concentrated along specific sections of the Jihe East Expressway, Guangshen Expressway, Qingping Expressway, and Nanguang Expressway. In comparison with the initial spatial distribution of the 150 cameras, the optimized traffic incident detection is more strategically focused on both location and incident type, thereby mitigating the impact of random and sporadic occurrences. This optimization highlights the distinct spatiotemporal characteristics of various types of traffic incidents. Consequently, in practical applications with limited computational resources, the optimized configuration method enables a scientifically accurate allocation of resources across spatiotemporal dimensions.

## 5. Conclusions

This paper addresses the challenges of urban road hazard management, particularly the current shortage of traffic police resources. It proposes an optimized traffic event detection configuration model using road surveillance cameras within the constraints of limited computing power. The proposed method leverages historical data and prior knowledge to calculate a weighted event feature value for each camera, serving as its detection efficiency. A cyclic elimination mechanism is introduced to eliminate inefficient cameras, dynamically adjust camera resource allocation and enhance overall detection performance. The case study analysis of the optimized configuration reveals that the location distribution, coverage, and types of anomaly warnings in the optimized traffic event detection are more strategically targeted, resulting in detection efficiency improvements of 40%, 28%, 17%, and 28% across different time windows. Additionally, the model exhibits a higher average load degree, reduced load balance degree, and higher computational resource utilization. It effectively avoids issues such as unreasonable computing power allocation, resource wastage, and inadequacy inherent in existing scheduling methods, thereby facilitating dynamic resource allocation for camera resources. This study demonstrates the feasibility and effectiveness of the optimization model, offering a novel approach that enhances cost-efficiency and performance in road accident prevention and hazard warning in the era of artificial intelligence. The model is suitable for periodic and dynamic scheduling in smart transportation systems, serving as a valuable reference for practical application.

## Figures and Tables

**Table 1:** Weight values for different types of traffic incidents.

Incident Type	Pedestrians on Highways	Abnormal Parking	Traffic Congestion	Non-Motorized Vehicles on Highways
Weight	0.42	0.24	0.15	0.19

**Table 2:** Data collected and detection efficiency scores for each camera location from 0:00 to 6:00, from 1 July 2023 to 14 July 2023.

Camera Location	Pedestrians on Highways	Abnormal Parking	Traffic Congestion	Non-Motorized Vehicles on Highways	Detection Efficiency
1	4	15	10	6	7.92
2	0	6	8	4	3.4
3	5	26	24	8	13.46
4	0	5	8	3	2.97
5	1	4	1	2	1.91
…	…	…	…	…	…

**Table 3:** Comparison of the overall efficiency of traffic incident detection before and after optimization.

Time Window	Before Optimization	After Optimization	Improvement
00:00–06:00	0.84	1.18	40%
06:00–12:00	2.26	2.89	28%
12:00–18:00	3.36	3.93	17%
18:00–24:00	1.06	1.36	28%

**Table 4:** Overall performance indicators of traffic events.

Time Window Interval	Overall Efficiency of Traffic Incident Detection (*f*)	Average Load Degree (*U*)	Load Balance Degree ($\bar{R}$)	Computational Resource Utilization (σ)
00:00–06:00	1.18	3.45	7.44	97.50%
06:00–12:00	2.89	8.11	14.57	98.17%
12:00–18:00	3.93	11.68	35.73	94.83%
18:00–24:00	1.36	4.29	21.40	97.33%

**Table 5:** Comparison of traffic event detection computing power scheduling methods.

Method Name	Average Load Degree (*U*)	Load Balance Degree ($\bar{R}$)	Computational Resource Utilization (σ)
Static Round-Robin Scheduling	4.25	37.43	77.22%
Weighted Round-Robin Scheduling	5.78	25.32	89.67%
Priority Round-Robin Scheduling	5.52	29.65	85.34%
Heuristic Adaptive Scheduling	6.88	19.78	96.96%

## Data Availability

The raw/processed data required to reproduce these findings cannot be shared at this time as the data also form part of an ongoing study.
